# P2X7 Receptors as a Therapeutic Target in Cerebrovascular Diseases

**DOI:** 10.3389/fnmol.2020.00092

**Published:** 2020-06-18

**Authors:** Abraham J. Cisneros-Mejorado, Alberto Pérez-Samartín, María Domercq, Rogelio O. Arellano, Miroslav Gottlieb, Friedrich Koch-Nolte, Carlos Matute

**Affiliations:** ^1^Instituto de Neurobiología, Universidad Nacional Autónoma de México, Juriquilla, Mexico; ^2^Achucarro Basque Center for Neuroscience, Departamento de Neurociencias, Universidad del País Vasco, CIBERNED, Leioa, Spain; ^3^Institute of Neurobiology, Slovak Academy of Sciences, Kosice, Slovakia; ^4^Department of Immunology, University Hospital, Hamburg, Germany

**Keywords:** ATP, pannexin-1, ischemia, neuron, oligodendrocyte

## Abstract

Shortage of oxygen and nutrients in the brain induces the release of glutamate and ATP that can cause excitotoxicity and contribute to neuronal and glial damage. Our understanding of the mechanisms of ATP release and toxicity in cerebrovascular diseases is incomplete. This review aims at summarizing current knowledge about the participation of key elements in the ATP-mediated deleterious effects in these pathologies. This includes pannexin-1 hemichannels, calcium homeostasis modulator-1 (CALHM1), purinergic P2X7 receptors, and other intermediaries of CNS injury downstream of ATP release. Available data together with recent pharmacological developments in purinergic signaling may constitute a new opportunity to translate preclinical findings into more effective therapies in cerebrovascular diseases.

## Introduction

Cerebrovascular diseases (CVDs) are referred to as a group of conditions that eventually lead to a reduction of blood supply to the brain as a consequence of a blockage (thrombosis or atherosclerosis), malformation (aneurysm), hemorrhage, or transient ischemia. In all instances, a decrease in oxygenation and nutrient supply ultimately leads to brain damage. Cerebrovascular diseases, principally stroke, constitute the second leading cause of death in adults worldwide and are major contributors to disability and reduced quality of life (Truelsen et al., 2000), so it is important to know the mechanisms behind this damage to reduce their consequences with the design and use of therapies against these diseases.

At the cellular level, damaged neurons and glial cells during and after stressful events in CVDs release glutamate into the extracellular space, which finally induces cytosolic Ca^2+^ overload and excitotoxicity (Braun et al., 1998; Jurányi et al., 1999; Melani et al., 2005; Takeuchi et al., 2006; for reviews, see also Rossi et al., 2007; Yenari et al., 2010). More recently, ATP was also defined as a potent excitotoxic signal to oligodendrocytes and neurons (Matute et al., 2007; Domercq et al., 2010; Cisneros-Mejorado et al., 2015b). In addition to ATP being among the molecules that are released by cell damage, recent evidence suggests that ATP acts as a damage-associated molecular pattern (DAMP) to initiate the innate immune response, induce pro-inflammation, and contribute to progressive neurological injury (Braun et al., 2017). Moreover, the massive increase in the cytosolic concentration of Ca^2+^ is due in part to the overactivation of P2X7 receptors, a nonselective ligand-gated cation channel expressed at the cell surface of various cell types and activated by extracellular ATP. In this review, we summarize the state of the art regarding the P2X7 receptor role in cerebrovascular damage and its possible use as a therapeutic target. In addition, we discuss its relationship with other molecular agents, such as pannexins (Panxs) and calcium-permeable channels, representing all together a pathological orchestrated cluster that can contribute to the onset of tissue damage and its propagation in CVDs.

## P2X7 Receptors Are Major Mediators of Tissue Damage in Cerebrovascular Diseases

**Figure 1:**
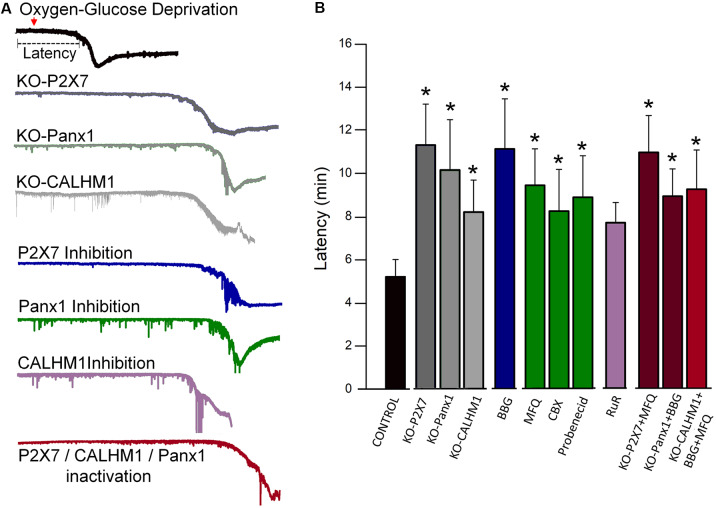
Blockade or genetic ablation of P2X7 receptors, pannexin-1 (Panx1), or calcium homeostasis modulator-1 (CALHM1) channels delays ischemic ionic currents in acute cortical slices. **(A)** Representative electrophysiological recordings in whole-cell configuration (holding potential at −80 mV), of ischemic (oxygen-glucose deprivation, OGD) ionic currents in cortical neurons in acute brain slices from wild-type mice as well as from either P2X7, Panx1, or CALHM1 KO mice in the presence or absence of inhibitors of these channels. **(B)** Histogram showing the latency of the onset of ischemic ionic currents following OGD in the absence (Control) or in the presence of P2X7 receptor antagonist Brilliant Blue G (BBG, 50 nM); Panx1 inhibitors mefloquine (MFQ, 100 nM), carbenoxolone (CBX, 100 μM), and probenecid (1 mM); and CALHM1 inhibitor ruthenium red (RuR, 20 μM). Similar increased latency values were observed in neurons from acute slices obtained from P2X7 receptor, Panx1, and CALHM1 knockout mice (KO-P2X7, KO-Panx1, and KO-CALHM1, respectively). Concomitant blockade of more than one target (red columns) did not result in further delay of the post-anoxic current. All the data included in this graph was published earlier (Cisneros-Mejorado et al., 2015a; Cisneros-Mejorado et al., 2018; *p < 0.05 vs. control).

Energy deprivation after stroke causes anoxic and irreversible depolarization. These events subsequently lead to massive release of excitatory neurotransmitters, including glutamate and ATP (Braun et al., 1998; Jurányi et al., 1999; Melani et al., 2005; Rossi et al., 2007), the latter causing neuronal and glial cell death through P2X7 receptor activation (Matute et al., 2007; Domercq et al., 2010; Arbeloa et al., 2012). However, the mechanisms of deleterious ATP release during brain ischemia are only partially known (Dale and Frenguelli, 2009; Cisneros-Mejorado et al., 2015b). Thus, during ischemia, the lack of oxygen causes a reduction in ATP production with an ensuing failure of plasma membrane ion pumps and loss of ion concentration homeostasis, all of which can finally lead to activation of Panx1 and calcium homeostasis modulator-1 (CALHM1) and the subsequent release of ATP (Cisneros-Mejorado et al., 2015b). These events lead to sustained activation of P2X7 receptors and pore formation with ensuing further ATP release that together creates a vicious circle, resulting in enhanced ATP-mediated excitotoxicity (Cisneros-Mejorado et al., 2015b). Indeed, pharmacological blockade or gene ablation of P2X7, Panx1, and CALHM1 results in substantial delayed post-anoxic depolarization following oxygen-glucose deprivation (OGD) and reduced brain tissue damage after transient middle cerebral artery occlusion (MCAO; Cisneros-Mejorado et al., 2015a, 2018; Figure 1).

In addition to neuronal and oligodendroglial excitotoxicity, activation of P2X7 receptors triggers the formation of the inflammasome, a multiprotein complex that mediates the release of cytokines, such as IL-1β, IL-18, and IL-33 (Giuliani et al., 2017; Baroja-Mazo et al., 2013), which can expand the initial ischemic damage.

P2X7 receptors are expressed in neurons and glia (see Table 1). Neurons express P2X7 receptors (Yu et al., 2008; Díaz-Hernández et al., 2009; Miras-Portugal et al., 2017; but see also Illes et al., 2017), and its blockade prevents ATP excitotoxicity and reduces the damage in models of both *in vivo* and *in vitro* ischemia (Arbeloa et al., 2012; Cisneros-Mejorado et al., 2015a). Likewise, oligodendrocytes, which are the major cellular component of white matter in the CNS, can undergo direct ATP-mediated excitotoxicity *via* activation of P2X7 receptors expressed in their membrane (James and Butt, 2001; Agresti et al., 2005; Matute et al., 2007; Yu et al., 2008; Domercq et al., 2010). Indeed, P2X7 receptors mediate ischemic damage to oligodendrocytes in culture and in optic nerve explants (Domercq et al., 2010). Moreover, oligodendrocyte precursor cells (OPCs) also express P2X7 receptors that contribute to periventricular white matter during perinatal hypoxic-ischemic injury as this condition is attenuated with selective antagonists of these receptors (Wang et al., 2009).

**Table 1:** P2X7 receptor expression in the central nervous system.

Cell type	Preparation	Techniques	References
Neuron	Mouse brain	Ca^2+^ imaging, IHH,	Díaz-Hernández et al. (2009)
		qPCR, WB	Miras-Portugal et al. (2017)
	Rat Brain	*in situ* hybridization	Yu et al. (2008)
Oligodendrocyte lineage	Rat forebrain OPCs *in vitro*	WB, Ca^2+^ imaging	Agresti et al. (2005)
	Rat optic nerve OLs *in situ*	Ca^2+^ imaging	James and Butt (2001)
	Rat brain OLs	qPCR	Yu et al. (2008)
	Rat and human optic nerve OLs	IHH, WB, Ca^2+^ imaging, electrophysiology	Matute et al. (2007); Domercq et al. (2010)
Microglia	Rat and mice brain	WB, IHH, *in situ* hybridization	Collo et al. (1997)
	Rat brain *in vitro*	Ca^2+^ imaging, electrophysiology	Visentin et al. (1999)
	Rat brain *in vitro*	Cytokine reléase, fluorimetry	Hide et al. (2000)
	Rat brain *in vitro*	IHH, fluorimetry	Verderio and Matteoli (2001)
	Mouse brain *in vitro*	Electrophysiology, fluorimetry	Chafke et al. (2002)
Astrocytes	Rat brain *in vitro*	Ca^2+^ imaging	Ballerini et al. (1996)
	Astrocyte cell line	Ca^2+^ imaging	Sun et al. (1999)
	Rat brain *in vitro*	IHH, chemokine signaling	Panenka et al. (2001)
	Rat brain after lesion	IHH	Franke et al. (2001)
	Rat brain	IHH	Kukley et al. (2001)
	Rat optic nerve *in situ*	Ca^2+^ imaging	James and Butt (2001)
	Rat spinal cord	Ca^2+^ imaging, ATP release	Suadicani et al. (2006)

*IHC, immunohistochemistry; OL, oligodendrocyte; OPCs, oligodendrocyte progenitor cells; qPCR, quantitative PCR; WB, western blot*.

On the other hand, microglia express P2X7 receptors (Ferrari et al., 1996; Collo et al., 1997; Visentin et al., 1999; Sanz and Di Virgilio, 2000; Hide et al., 2000; Verderio and Metteoli, 2001; Chafke et al., 2002) that can promote their activation and proliferation (Bianco et al., 2006; Monif et al., 2009). This can indirectly cause neurotoxicity by stimulating the production of reactive oxygen species (Bartlett et al., 2013) as well as the release of pro-inflammatory cytokines (Suzuki et al., 2004; Shieh et al., 2014). Interestingly, P2X7 receptors in primary adult human microglia kept in culture modulate key components of innate immunity (Janks et al., 2018). Finally, P2X7 receptors are present in astrocytes (Ballerini et al., 1996; Sun et al., 1999; Panenka et al., 2001; Franke et al., 2001; James and Butt, 2001; Kukley et al., 2001), whereby they can raise intracellular Ca^2+^ concentration and ATP release (Suadicani et al., 2006) as well as contribute to non-vesicular glutamate release (Duan et al., 2003). Table 1 summarizes the evidence supporting the expression of P2X7 receptors in neurons and glia.

Together, these data indicate that activation of P2X7 receptors can activate deleterious signals after ischemia by altering Ca^2+^ homeostasis and promoting the release of pro-inflammatory cytokines as well as causing oxidative stress. Accordingly, P2X7 blockade reduces tissue damage in experimental models of CVDs. Thus, Brilliant Blue G (BBG), a P2X7 selective antagonist, attenuates the extent of brain damage following transient MCAO (Arbeloa et al., 2012; Cisneros-Mejorado et al., 2015a). Similarly, BBG treatment ameliorates neuronal apoptosis in an experimental subarachnoid hemorrhage model (Chen et al., 2013). Moreover, three different P2X7 antagonists (BBG, A0438079, and OxATP) significantly increase survival rates and reduce cognitive deficits and cell death in transient global ischemia-reperfusion injury (Chu et al., 2012). Notably, P2X7 receptor attenuated glial activation and inflammatory cytokine overexpression in the hippocampus (Chu et al., 2012). Table 2 sums up the protective effects of the inhibition or deletion of P2X7 described above.

**Table 2:** Protective effects of blocking of P2X7 receptors.

Antagonist	Model	Effects	References
Brilliant Blue G	OGD, MCAO	Decreases infarct size	Arbeloa et al. (2012)
		Protects from neuronal death	Chu et al. (2012)
		Relieves neurological symptoms	Cisneros-Mejorado et al. (2015a)
	*in vitro*, EAE	Promotes oligodendrocyte survival, protects myelin, ameliorates neurological symptoms	Matute et al. (2007)
	OGD *in vitro* and *in situ*	Promotes oligodendrocyte survival, protects myelin	Domercq et al. (2010)
	Perinatal hypoxia-ischemia	Reduces white matter injury	Wang et al. (2009)
	experimental subarachnoid hemorrhage	Ameliorates function and reduces neuronal apoptosis	Chen et al. (2013)
A0438079	MCAO	Relieves neurological symptoms	Chu et al. (2012)
		increase survival rates	
		Attenuate inflammation	
	OGD in slices and culture	reduce postanoxic depolarization	Cisneros-Mejorado et al. (2015a)
OxATP	MCAO	Reduces mortality	Chu et al. (2012)
	*in vitro*, EAE	Promotes oligodendrocyte survival, Protects myelin, ameliorates neurological symptoms	Matute et al. (2007)
Nanobodies	*in vivo* glomerulonephritis	Ameliorates experimental glomerulonephritis in mice	Danquah et al. (2016)

*EAE, experimental autoimmune encephalomyelitis; MCAO, transient middle cerebral artery occlusion; OGD, oxygen-glucose deprivation*.

## Activation and Biophysical Properties of P2X7 Receptors in Neurons and Glia

P2X7 receptors have two possible states of conductance. First, low concentrations of agonist (ATP or BzATP) induce activation of a nonselective monophasic conductance allowing monovalent (Na^+^, K^+^) and divalent (Ca^2+^) cation influx and plasma membrane depolarization (North, 2002). In addition, after prolonged exposure to high agonist concentrations (exposure to high agonist concentrations >1 mM ATP, >30 μM BzATP) P2X7 receptors can form a large nonselective pore allowing the passage of organic cations and molecules of up to 900 Da and the leakage of metabolites, including ATP (North, 2002; Yan et al., 2010), as shown in astrocytes (see above; Suadicani et al., 2006) and microglia (Ferrari et al., 1997). Although it is not clear whether oligodendrocytes leak ATP by this mechanism, there is evidence showing that glucocorticoids increase hemichannel activity in these cells in a P2X7R-dependent manner, suggesting a possible formation of a nonselective pore in these conditions (Maturana et al., 2017). Interestingly, P2X7 receptors in cultured cortical neurons form a large pore only at very high concentrations of BzATP and following a prolonged (10–40 s) exposure to the agonist (Cisneros-Mejorado et al., 2015b) although, in astrocytes *in vitro* or HEK cells expressing P2X7 receptors, the large pore formation occurs more readily (Yan et al., 2010). Macropore formation cannot be due to receptor pore dilation *per se* because the single-channel current amplitude and permeation characteristics remain constant (Riedel et al., 2007; Pippel et al., 2017; Di Virgilio et al., 2018). Thus, it appears that additional mechanisms must underlie the opening of the macropore (see Braun et al., 2017). This suggests that large pore formation in cultured neurons depends not only on prolonged stimulation of the P2X7 receptor. Alternatively, a role in ATP release has also been assigned to membrane channels formed by Panx1, which are large-pore ion channels with broad expression in the CNS (MacVicar and Thompson, 2010). Panx1 is permeable to molecules up to 900 Da and directly mediates ATP release (Locovei et al., 2007; Iglesias et al., 2009). The mechanisms by which P2X7 receptors and Panx1 are involved in pore formation are not well defined. However, the evidence indicates in astrocytes, for example, that Panx1 channels are activated after P2X7 receptor stimulation (Iglesias et al., 2009) or on concomitant membrane depolarization to induce Panx1 opening as reported earlier (Locovei et al., 2007). Thus, Panx1 can be activated following Ca^2+^ influx *via* P2X7 receptors along or in conjunction with subsequent Ca^2+^-induced Ca^2+^ mobilization from intracellular stores (North, 2002; Locovei et al., 2006). These findings suggest that P2X7 receptors and Panx1 act synergistically, at least in the CNS, because, in other systems, pore formation after prolonged activation of the P2X7 receptor does not occur through Panx1 channels (Qu et al., 2011; Alberto et al., 2013). One of the best characterized aspects of P2X7 receptor function is its ability to activate indirectly the NACHT, LRR, and PYD domains containing protein 3 (NLRP3) inflammasome in macrophages, thereby initiating caspase-1-mediated IL-1β; processing and release and ultimately inducing macrophage pyroptotic cell death (Chen et al., 2014). In the brain, exogenous ATP induces NLRP3 inflammasome activation in astrocytes and microglia along with an increase in IL-1β production that activates caspase-1 and amplification of the stress response (Murphy et al., 2012). Consistent with this idea, stressed glia increases connexin 43 (Cx43) and Panx1 hemichannel activity in microglia and astrocytes of adult mice (Orellana et al., 2015). Moreover, exposure to high levels of glucocorticoids during gestation induces long-lasting neuroinflammation and activates the inflammasome in hippocampal oligodendrocytes of mouse offspring (Maturana et al., 2017). Thus, oligodendrocytes of control pups showed expression of inflammasome components (NLRP3, ACS, and caspase-1), and their levels were increased by prenatal administration of dexamethasone, a synthetic glucocorticoid. These cells also showed high levels of IL-1β; and TNF-α; in accordance with activation of the inflammasome. Notably, oligodendrocytes showed increased levels of the P2X7 receptors and Panx1, molecules associated with inflammasome activation (Maturana et al., 2017). However, it is not known whether glucocorticoids also modify the activation properties of the P2X7/Panx1 axis in neurons, a feature that might be relevant in CVDs.

## P2X7 Receptors Are Involved in Ischemic Pre- and Post-Conditioning Effects

Brief, non-harmful, ischemic preconditioning can confer tolerance and protection from subsequent cerebrovascular damage. Thus, preconditioning attenuates damage in models of cerebral ischemia associated with oxidative stress and glutamate excitotoxicity (Jachova et al., 2019). Although the mechanisms of this phenomenon are unclear, recent data suggest that P2X7 receptors have a role in ischemia preconditioning as this is ineffective in P2X7 receptor knockout mice (Hirayama et al., 2015). In turn, after preconditioning, P2X7 receptors in astrocytes can orchestrate the initiation of neuroprotective cascades including those mediated by HIF-1α; Hirayama et al., 2015). Indeed, P2X7 receptor expression itself is increased in astrocytes and followed by an elevation of hypoxia-inducible factor (HIF)-1α in these cells after preconditioning using an MCAO model in mice (Hirayama et al., 2015). Moreover, inhibiting the astroglial metabolism with fluorocitrate abolished the induction of ischemic tolerance, which strongly suggests that astrocytes play an essential role in its inception (Hirayama et al., 2015).

In addition, hypoxic preconditioning protects cultured neurons against hypoxic stress *via* tumor necrosis factor-α; TNF-α; Jie Liu et al., 2000; Ruscher et al., 1998). Notably, the release of TNF-α appears to be dependent on the P2X7 receptor because microglia treated with BzATP in neuron-microglia cultures leads to significant reduction in glutamate-induced neuronal cell death, and either TNF-α-converting enzyme inhibitor or anti-TNF-α IgG readily suppresses this protective effect (Suzuki et al., 2004). These findings provide evidence that, as in astrocytes, P2X7 receptors in microglia contribute to brain ischemic tolerance though different mechanisms.

The above data argues in favor of a dual role of P2X7 receptors in CVDs with a protective edge during mild ischemia (preconditioning) and a deleterious excitotoxic role in more severe ischemia. This duality adds further complexity to the development of effective therapies to prevent ischemic injury by promoting, for example, preconditioning with P2X7 receptor agonists and/or allosteric modulators while having at hand antagonists as neuroprotectants to limit the extension of the ischemic core into the penumbra once stroke occurs. To define the parameters that limit this double-edged behavior of P2X7 receptors constitutes a major challenge in experimental ischemia as a previous step for an effective translation into CVDs patients.

On the other hand, ischemic post-conditioning may be more amenable and effective for therapeutic use. Thus, repetitive short periods of OGD alternated with reperfusion after prolonged OGD attenuates neuronal apoptosis by increasing Bcl-2 expression while reducing Bax levels and overexpressing heat-shock protein 70 (HSP70; Zhao et al., 2014). Intriguingly, proteomic and functional characterization of the P2X7 receptor signaling complex showed that HSP70 co-immunoprecipitates with P2X7 receptors (Kim et al., 2001). This finding strongly suggests an interaction between HSP70 and P2X7 receptors; however, a direct link between P2X7 activity and post-conditioning neuroprotection is still missing.

## Therapeutic Potential of P2X7 Receptor in Cerebrovascular Diseases

All the above evidence suggests that P2X7 receptor activation or blockade is involved in the onset and propagation of tissue damage as well as in neuroprotection and neuroinflammation in CVDs. Therefore, a thorough understanding of the upstream events leading to P2X7 receptor activation along with subsequent downstream signaling cascades they trigger will eventually allow identifying new cellular and molecular targets amenable for the design of novel drugs to use in clinical studies.

P2X7 receptor agonists may facilitate ischemia preconditioning by promoting the release of protective factors and signals in neurons and glia that ultimately attenuate major damage (Hirayama et al., 2015). Alternatively, specific P2X7 agonists may enhance the phagocytic capacity of microglia as shown in a model of phagocytic function in fresh human monocytes without promoting pore formation, thus avoiding unwanted side effects, such as excitotoxicity or enhanced neuroinflammation (Ou et al., 2018). This novel role of the P2X7 receptor as a scavenger receptor in microglia/macrophages and possibly in other cells in the CNS creates new pharmacological possibilities as it is not affected by potent selective P2X7 receptor antagonists, and its phagocytic function has features distinct from its pore function (Ou et al., 2018). Therefore, differential drug targeting both on P2X7 pore formation and P2X7-mediated phagocytosis has a great potential as a single or combined treatment in CVDs.

In addition to agonists and antagonists, other P2X7 receptor ligands may have therapeutic value. This includes positive allosteric modulators, such as clemastine, an anti-allergy drug, which binds extracellularly to P2X7 receptors and potentiates their ATP-sensitivity while it increases the release of IL-1β; from lipopolysaccharide-primed macrophages, thus modulating native immune responses (Nörenberg et al., 2011). In turn, clemastine may also favor myelination of damaged myelin and the rescue of behavioral changes that occur after stroke (Liu et al., 2016; Cohen and Tesar, 2017). Likewise, ginsenosides of the protopanaxdiol series potently activate P2X7 receptors, leading to an increase of sustained calcium ion influx in mouse macrophages that may account for their reported immune modulatory actions *in vivo* (Helliwell et al., 2015).

Discovering new uses for approved drugs acting at P2X7 may provide the quickest possible transition from bench to bedside in CVDs. Thus, A-740003 (N-(1-[(cyanoimino)(5-quinolinylamino) methyl] amino-2,2-dimethylpropyl)-2-(3,4-dimethoxyphenyl)acetamide), a competitive antagonist of P2X7 receptors, produces significant antinociception in animal models of neuropathic and inflammatory pain (Honore et al., 2006) and has been evaluated for neuroinflammation (Janssen et al., 2014). On the other hand, AZD9056, an adamantane amide that was discovered through a program designed to identify potent and selective P2X7 antagonists, provides a significant inhibition of ATP-induced IL-1β release in monocytes ex vivo, suggesting that circulating leucocytes were blocked by P2X7 (Keystone et al., 2012). AZD9056 is well tolerated and induces statistically significant changes in parameters of clinical relevance; however, it failed to ameliorate symptoms in patients with rheumatoid arthritis, an immunologically mediated disease in which cytokines are key regulatory molecules. Similarly, high throughput screening of a compound library provided an attractive lead compound with modest P2X7 receptor antagonist potency and high selectivity against a panel of receptors and channels (Duplantier et al., 2011). Multi-parameter optimization led to a potent P2X7 antagonist, CE-224,535, which was advanced to clinical studies for the treatment of rheumatoid arthritis (Duplantier et al., 2011). This compound is currently under scrutiny for others brain diseases and has therapeutic potential in CVDs.

Another interesting approach in the development of biologics targeting P2X7 receptors are antibodies and nanobodies that antagonize or potentiate gating of P2X7. Their potential advantages over small-molecule drugs include high specificity, lower off-target effects, and tunable *in vivo* half-life (for a recent review see Koch-Nolte et al., 2019). Therapeutic antibodies are commonly injected systemically to maximize delivery; however, they can be also administered as aerosols to treat respiratory tract and lung diseases (Van Heeke et al., 2017). In addition, genetic fusion of P2X7-specific biologics to binding modules may enable targeting of specific cell subsets; besides, directly modulating P2X7 function, antibodies can also initiate specific depletion of P2X7-expressing cells (Koch-Nolte et al., 2019). Furthermore, adeno-associated viral vectors can be used to express P2X7-specific antibodies *in vivo* to achieve long-lasting biological effects and enable modulation of the function of P2X7-expressing immune cells *via* encoded transgenic RNA or proteins (Koch-Nolte et al., 2019). Indeed, functional antibodies and nanobodies have already shown promising therapeutic benefit in animal models of sterile inflammation (Menzel et al., 2018).

## Conclusions

The present review provides an outlook about the role of P2X7 receptors in CVDs and their dual function as a cationic channel and as a precursor of large pore formation. It summarizes a wealth of evidence demonstrating that inhibition of P2X7 receptors promotes neuron and glia protection against brain injury and that they can be essential for the release of cell-supporting factors. At the same time, these receptors are also relevant to preconditioning and post-conditioning and, therefore, emerge as possible targets to attenuate tissue damage in CVDs and modulate neuroinflammation to constrain the expansion of the core lesion into the penumbra.

New, groundbreaking research on therapeutic targeting on P2X7 receptors is constantly being made available. A notable example is the relatively recent discovery of a structural basis for subtype-specific inhibition, which provides novel mechanistic insights to facilitate the development of P2X7-specific drugs for treating human diseases (Karasawa and Kawate, 2016). Moreover, new data on the structural and functional properties, in combination with cell-based functional studies, suggest that the P2X7 receptor itself constitutes a lipid-composition-dependent, dye-permeable pore, whose opening is facilitated by palmitoylated cysteines near the pore-lining helix (Karasawa et al., 2017).

**Figure 2:**
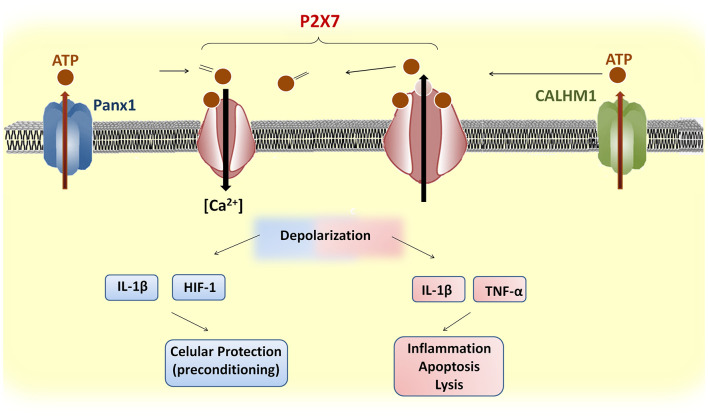
Upstream and downstream events of P2X7 receptor activation in brain cells. ATP release through Panx1 or CALHM1 activates P2X7 receptors, which induces influx of Ca^2+^. The prolonged stimulation of P2X7 receptors induces pore formation and further ATP release. Brief depolarization during mild cerebrovascular diseases (CVDs) may promote the release of protective factors, such as hypoxia-inducible factor (HIF)-1, that confer cellular protection against subsequent ischemic stimuli. In contrast, severe CVDs trigger a sustained P2X7 receptor depolarization and the release of pro-inflammatory cytokines that cause apoptosis or lysis.

In summary, P2X7 receptors contribute to neurotransmission and glia signaling using Ca^2+^ as a key second messenger (Figure 2) although the precise mechanisms mediating their effects in neurons and glial cells are still unclear. In mild CVDs, P2X7 receptors are involved directly or indirectly in preconditioning or post-conditioning by conditions by promoting the release of protective factors, such as HIF-1 (Hirayama et al., 2015), resulting in a pro-survival cascade against subsequent harmful events in neurons and possibly in oligodendrocytes. Finally, in severe CVDs, P2X7 receptors promote pore formation, thus allowing the efflux of large molecules including ATP and Ca^2+^ influx causing cytosolic Ca^2+^ overload and generating a detrimental feedback loop that ultimately results in neuronal and oligodendroglial death with the consequent demyelination along with astrocytic and microglial activation as well as pro-inflammatory cytokine release. Further work is warranted to elucidate the exact underlying mechanisms of the P2X7 receptor in the pathophysiology of CVDs and to shed light on therapies that simultaneously target multiple cell types and mechanisms of injury in these diseases.

## Author Contributions

AC-M and CM conceived and described the initial draft of the manuscript. AC-M prepared graphic material. AP-S, MD, RA, MG, and FK-N contributed numerous comments and suggestions to the final manuscript.

## Conflict of Interest

The authors declare that the research was conducted in the absence of any commercial or financial relationships that could be construed as a potential conflict of interest.
